# Fracture fixation in the hand and wrist: A 16-year population-based study of 56 163 patients from the Swedish National Patient Register

**DOI:** 10.1371/journal.pone.0330116

**Published:** 2025-09-03

**Authors:** Viktor Schmidt, Elsa Pihl, Cecilia Mellstrand Navarro, Michael Axenhus

**Affiliations:** 1 Danderyd Hand and Wrist Initiative, Danderyd Hospital, Stockholm, Sweden; 2 Department of Orthopaedic Surgery, Danderyd Hospital, Stockholm, Sweden; 3 Department of Clinical Sciences at Danderyd Hospital, Karolinska Institutet, Stockholm, Sweden; 4 Department of Clinical Science and Education Södersjukhuset, Karolinska Institutet, Stockholm, Sweden; Showa University, JAPAN

## Abstract

Hand and wrist fractures are among the most common orthopaedic injuries, with a growing trend toward surgical treatment. However, large-scale data on regional variations and treatment trends remain limited. This population-based study analyses the incidence, trends, and regional variations in hand and wrist fracture fixation with plates and screws in Sweden from 2008 to 2023, with predictive modelling for future trends. A total of 56,163 patients aged ≥15 years underwent fixation (code NDJ69). Southern regions, including Skåne and Halland, had the highest fixation rates (>100/100,000), while northern areas like Norrbotten had significantly lower rates (<20/100,000). Women ≥65 years had the highest incidence. Predictive models indicate a continued increase in procedures, particularly among women aged ≥65, through 2035. These findings highlight regional disparities and the ongoing shift towards surgical treatment in older populations, emphasizing the need for optimized treatment strategies to ensure equitable access to care.

## Introduction

Wrist fractures are the most common fractures in adults [1,2] and their incidence is increasing worldwide [2,3]. Together with hand fractures, they account for up to 30% of emergency department visits [3–5]. Treatment decisions are based on both radiological findings and patient-specific factors. However, there is ongoing debate about the optimal type of surgery and the ideal timing [6–8]. Over the 21^st^ century, surgical intervention has become increasingly preferred [9]. In displaced fractures, surgery more reliably restored anatomical alignment compared with nonsurgical treatment [10,11], which may be crucial for achieving favourable clinical outcomes [12]. Furthermore, early primary surgery appears to yield better hand function compared to delayed primary surgery [7,10].

Because definitions of acceptable radiological alignment vary, treatment recommendations differ both between and within countries [13–15].

Using large-scale data from the Swedish National Patient Register (NPR), this study aims to analyse demographic variations in incidence rates of fracture fixation—emphasising age and sex differences—evaluate regional disparities to identify high- and low-incidence of surgery areas; and forecast future trends to support healthcare planning.

## Methods

### Study design and setting

This population-based observational study utilizes open-access surgical data obtained from the NPR for the period between 2008 and 2023. The study complies with the RECORD guidelines [16]. The data used in this study is obtained from the website of the SNBHW and is publicly available for anyone to download and use.

### Healthcare system overview

Sweden’s National Health Service provides universal healthcare access to all residents, covering emergency care, general hospital services, and outpatient visits at no cost. Although private hospitals exist, fracture cases are universally treated in public facilities. Each resident is assigned a unique Swedish personal identification number that remains valid throughout their lifetime or until emigration [17]. This identifier is integral for interactions within both public and private healthcare systems and is linked to all national healthcare registers.

### Data source

The NPR is a comprehensive registry documenting healthcare data for patients treated within Sweden’s closed healthcare system [18]. The NPR has recorded inpatient care since 1964 (nationwide since 1987) and included specialized outpatient services starting in 2001. Updates were annual until 2021 but have been conducted monthly since June 2021, incorporating delayed or corrected data. The registry provides detailed records of surgical procedures, including geographic distribution, age, and sex of patients. All healthcare providers, both public and private, are required to report diagnoses and procedures. This includes ICD-10 diagnoses [19], (since 1994) and surgical procedure codes classified under the NOMESCO framework [20]. Data from all hand surgery and orthopaedic departments in Sweden are included in the NPR. The registry has been reported to provide data of high quality [21,22].

### Patient selection

The study considered individuals aged 15 years or older who underwent fracture fixation with a plate in the hand or wrist, as recorded in the NPR between January 1, 2008, and December 31, 2023. Only individuals with Swedish personal identification numbers were included.

### Study population

Inclusion criteria:

1. Individuals with residency in Sweden at one point during 1st of January 2008 and 31st of December 2023.
2. Individuals who were registered with a fracture fixation in the hand or wrist (NOMESCO code NDJ69).

Exclusion criteria:

1. Age <15 years.

### Statistical analysis

The data was extracted as incidence of surgical procedure per 100,000 inhabitants. Data were stratified by sex, age group, and region to facilitate trend comparisons over time. Incidence rates were controlled by a separate incidence calculation that was done by dividing the number of patients by the total population, as reported by Statistics Sweden [23]. Significant differences between sex in each age groups were assessed using the Student’s t-test. To predict future trends, regression analysis was employed, and we fitted exponential, linear, logarithmic, polynomial, and power regression models for each incidence trend. The year was used as the independent variable and incidence of fracture surgery was as the exposure. The assumptions underlying regression analysis were assessed to ensure model validity. Linearity was evaluated visually using scatter plots of residuals versus fitted values. Independence of residuals was checked using the Durbin-Watson statistic. Full stationarity testing not applicable due to the short time series. Model validation was performed by comparing the adjusted R² across models to ensure generalizability without overfitting. The regression analysis used only data from 2014 to 2023 to account for significant changes in treatment practice which were implemented before 2014. Predictive analysis was based on the best-fitting model, with 95% confidence intervals (CI) applied where relevant. A p-value of <0.05 was considered statistically significant. * indicate <0,05, ** < 0,01, ** < 0,001 and *** < 0,0001. Regional maps graphs were created in Illustrator 2025 (29.5.1).

### Ethical considerations

This study relied exclusively on open-access data (which is anonymized and aggregated to avoid small-cell risks) and was thus exempt from ethical review as Swedish law does not require ethical permit for the study of group level open access data.

## Results

**Fig 1:**
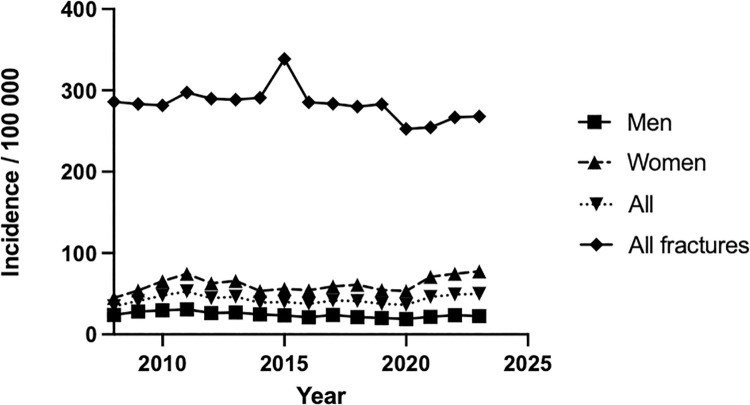
Trends in hand and wrist fracture fixation with plates and screws, 2008–2023. “All fractures” represent those treated with all modalities (non-operative included). The other three trend lines (Men, Women and All) represent only fixation using plates and screws.

A total of 56 163 patients were identified with the code NDJ69 during the study period. The overall incidence of fracture fixation increased steadily from 2008 to 2023. The annual incidence trends for fracture fixation with plates and screws from 2008 to 2023 show an increase in fracture fixation for women after 2020 compared to total amount of fracture diagnoses (Fig 1).

**Fig 2:**
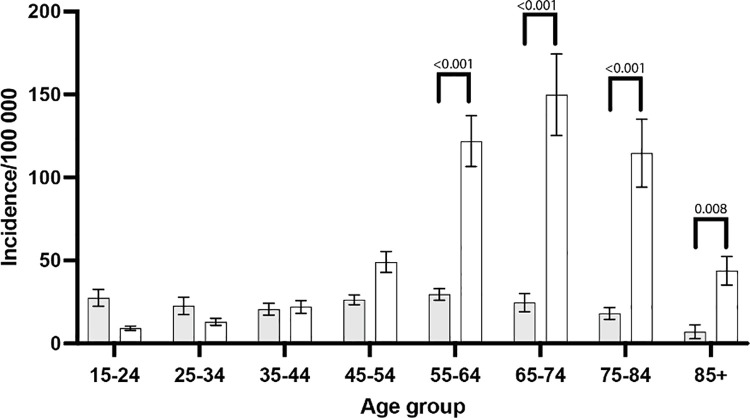
Age groups of patients who underwent fixation with plate for wrist or hand fractures. White indicates women, grey indicate men.

Age and sex distributions revealed distinct patterns. The incidence was highest in women aged ≥65 years. Younger patients, 15–64 years, showed relatively stable rates over the study period without notable differences between sexes (Fig 2).

**Fig 3:**
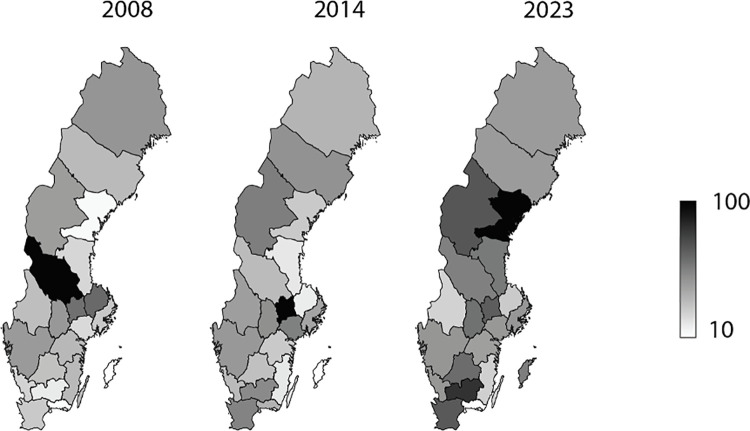
Regional variance in fracture fixation in the hand and wrist using plate and screws, comparison between 2008, 2014 and 2023. Incidence is marked as a colour gradient from 10 to 100 per 100,000 inhabitants.

Significant geographic disparities in procedure rates were noted. In 2023, significant geographic disparities in the metric were observed across Sweden. Northern regions like Västernorrlands län (95.7) and Jämtlands län (66.9) had notably high values, while southern areas such as Blekinge län (23.4) and Kalmar län (26.1) were considerably lower. Central regions like Stockholms län (45.4) aligned closely with the national average of 49.9. (Fig 3).

**Fig 4:**
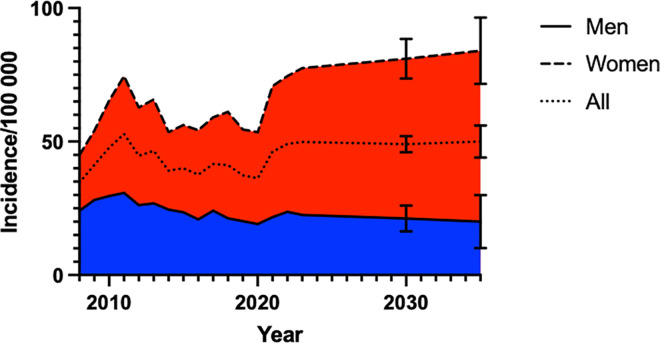
Future trend predication of hand and wrist fracture fixation.

Model fitting found a polynomial regression model of the 2^nd^ order to be best fit (S1 Table). Regression modelling indicated a continued rise in procedure incidence for women while men are expected to have a similar or slightly decreasing trend up until 2035 (Fig 4).

## Discussion

Our study reveals a significant increase in the utilization of plate and screw fixation for hand and wrist fractures in Sweden from 2008 to 2023, with a notable rise among women aged 65 and older. Regional disparities were observed, with southern regions like Skåne and Halland exhibiting higher incidence rates compared to northern areas such as Norrbotten. Predictive models suggest a continued annual increase of these procedures amongst women through 2035.

The increasing utilization of plate and screw fixation for hand and wrist fractures in Sweden reflects a global trend toward favouring internal fixation methods [9]. This shift is particularly evident among women aged ≥65, a demographic with a higher prevalence of osteoporosis, making them more susceptible to fractures [24]. The implementation of Sweden’s national guidelines in 2021, advocating for prompt surgical intervention for specific unstable distal radius fractures, seem to have significantly influenced clinical practice [25,26]. These guidelines recommend surgery within one week of injury to enhance patient outcomes amongst patients with high functional demands. The national guidelines are also partly radiologically based with certain criteria prompting surgery. Our study period, 2008–2023, encompasses the introduction of these guidelines, and while a direct causal relationship cannot be conclusively established, the observed increase in surgical interventions during this time suggests a correlation. Moreover, such guidelines might draw attention to surgical treatment of vital elderly patients who otherwise might have been subject to a conservative regime due solely to their chronological age, increasing equity along with the operative incidence, this might in turn lead to improved clinical outcomes [8,26].

The national guidelines aim to standardize treatment protocols across various patient demographics, ensuring equitable care and minimising unwarranted treatment variations. This standardization is expected to lead to more predictable outcomes and improved overall patient satisfaction. Implementing these guidelines necessitates adequate resources, including trained personnel and surgical facilities, to accommodate the increased demand for timely surgical interventions. However, the effectiveness of these guidelines may vary depending on the level of implementation at different hospitals [26]. Healthcare systems must ensure that resources are equitably distributed to prevent regional disparities. Educating patients about the benefits and risks associated with early surgical intervention is crucial. Shared decision-making should be encouraged, considering individual patient preferences, risks, and expected outcomes. National quality registries play a pivotal role in collecting data to evaluate the effectiveness of implemented guidelines and inform future revisions. Due to increasing volume of surgery, implant removal might become a future concern for healthcare providers while surgeries addressing complications such as correctional osteotomies might decrease.

Regional variations in procedure rates are notable, with southern regions such as Blekinge and Kalmar showing higher incidences compared to northern regions like Norrbotten. Several factors may contribute to these disparities. Southern Sweden***’***s higher population density and greater access to specialized orthopaedic services may facilitate more frequent surgical interventions. In addition, regional differences in medical tradition and adherence to national guidelines can influence treatment choices. Variations in age distribution, increasing number of older patients and the prevalence of osteoporosis across regions may also contribute to differing fracture rates and treatment decisions. These findings point to broader differences in healthcare delivery and utilization that warrant further study. In particular, the potential benefits of increased use of plate fixation amongst women is yet to be determined.

Research suggests that surgical variation primarily stems from differences in physician beliefs about the indications for surgery, as well as the degree to which patient preferences shape treatment decisions [27,28]. These factors, in turn, are influenced by the environmental variables discussed above.

The increasing adoption of plate and screw fixation for hand and wrist fractures in Sweden has significant clinical implications. The aim of early fixation is to enable favourable conditions for early mobilization, which is crucial in preventing joint stiffness and promoting functional recovery. However, the rise in these procedures necessitates that orthopaedic surgeons maintain proficiency in advanced fixation techniques as well as proper patient selection to ensure optimal outcomes. Although the incidence of surgical fixation has increased in Sweden, it remains unclear if this has led to increased patient satisfaction and clinical outcomes. Whether the implementation of a nation-wide guidelines has had positive healthcare effects remains unanswered and is an area for further study. It must also be mentioned that definitive treatment remains a clinical decision in which surgeons must considering factors like bone quality and fracture type, to determine the most suitable treatment approach.

### Limitations

The accuracy of the NPR depends on consistent and precise reporting from healthcare providers. Variations in coding practices, potential miscoding, transcription errors, underreporting, and missing data may influence data quality, as is typical in register-based studies. These variations could differ between regions. The specific procedural code NDJ69 represents open reduction and internal fixation of a fracture in the wrist or hand with plates and screws. It does not include other modalities or anatomical sites. Additionally, the observational nature of our study limits our ability to control for confounding factors such as patient comorbidities, socioeconomic status, and bone quality, which may influence treatment decisions and outcomes. The NPR primarily records procedural data and lacks detailed clinical outcomes, such as functional recovery and patient satisfaction, restricting our ability to assess the long-term effectiveness of the surgical interventions studied. Furthermore, the study does not account for patients treated non-surgically or with alternative methods, introducing potential selection bias. Future research should consider incorporating comprehensive patient information, employing advanced statistical methods to adjust for potential confounders, and conducting prospective studies to establish causal relationships and evaluate long-term outcomes.

## Conclusion

Our study reveals a significant increase in plate and screw fixation for hand and wrist fractures in Sweden from 2008 to 2023, particularly among women aged 65 and older. Southern regions like Blekinge and Kalmar exhibit higher incidence rates compared to northern areas such as Norrbotten. Predictive models indicate an annual increase in these procedures amongst women through 2035. The implementation of national guidelines in 2021, advocating for prompt surgical intervention for distal radius fractures, appears to have influenced these trends. Further research is warranted in order to determine if increased surgical fixation correlate to patient satisfaction and clinical outcomes.

## Supporting information

S1 Table Adjusted R2 values for tested regression models. (DOCX)

## References

[1] van Staa TP, Dennison EM, Leufkens HG, Cooper C. Epidemiology of fractures in England and Wales. Bone. 2001;29(6):517–22. doi: 10.1016/s8756-3282(01)00614-7 11728921

[2] Nellans KW, Kowalski E, Chung KC. The epidemiology of distal radius fractures. Hand Clin. 2012;28(2):113–25. doi: 10.1016/j.hcl.2012.02.001 22554654 PMC3345129

[3] MacIntyre NJ, Dewan N. Epidemiology of distal radius fractures and factors predicting risk and prognosis. J Hand Ther. 2016;29(2):136–45. doi: 10.1016/j.jht.2016.03.003 27264899

[4] Angermann P, Lohmann M. Injuries to the hand and wrist. A study of 50,272 injuries. J Hand Surg Br. 1993;18(5):642–4. doi: 10.1016/0266-7681(93)90024-a 8294834

[5] Larsen CF, Mulder S, Johansen AMT, Stam C. The epidemiology of hand injuries in The Netherlands and Denmark. Eur J Epidemiol. 2004;19(4):323–7. doi: 10.1023/b:ejep.0000024662.32024.e3 15180102

[6] Costa ML, Achten J, Rangan A, Lamb SE, Parsons NR. Percutaneous fixation with Kirschner wires versus volar locking-plate fixation in adults with dorsally displaced fracture of distal radius: five-year follow-up of a randomized controlled trial. Bone Joint J. 2019;101-B(8):978–83. doi: 10.1302/0301-620X.101B8.BJJ-2018-1285.R1 31362548 PMC6681675

[7] Mulders MAM, Walenkamp MMJ, van Dieren S, Goslings JC, Schep NWL. Volar plate fixation versus plaster immobilization in acceptably reduced extra-articular distal radial fractures. J Bone Joint Surgery. 2019;101(9):787–96. doi: 10.2106/jbjs.18.00693 31045666

[8] Saving J, Severin Wahlgren S, Olsson K, Enocson A, Ponzer S, Sköldenberg O, et al. Nonoperative treatment compared with volar locking plate fixation for dorsally displaced distal radial fractures in the elderly: a randomized controlled trial. J Bone Joint Surg Am. 2019;101(11):961–9. doi: 10.2106/JBJS.18.00768 31169572

[9] Mellstrand-Navarro C, Pettersson HJ, Tornqvist H, Ponzer S. The operative treatment of fractures of the distal radius is increasing: results from a nationwide Swedish study. Bone Joint J. 2014;96-B(7):963–9. doi: 10.1302/0301-620X.96B7.33149 24986952

[10] Sirniö K, Leppilahti J, Ohtonen P, Flinkkilä T. Early palmar plate fixation of distal radius fractures may benefit patients aged 50 years or older: a randomized trial comparing 2 different treatment protocols. Acta Orthop. 2019;90(2):123–8. doi: 10.1080/17453674.2018.1561614 30669897 PMC6461076

[11] Ochen Y, Peek J, van der Velde D, Beeres FJP, van Heijl M, Groenwold RHH, et al. Operative vs nonoperative treatment of distal radius fractures in adults: a systematic review and meta-analysis. JAMA Netw Open. 2020;3(4):e203497. doi: 10.1001/jamanetworkopen.2020.3497 32324239 PMC7180423

[12] Schmidt V, Gordon M, Tägil M, Sayed-Noor A, Mukka S, Wadsten M. Association between radiographic and clinical outcomes following distal radial fractures. J Bone Joint Surgery. 2023;105(15):1156–67. doi: 10.2106/jbjs.22.01096 PMC10377255 37172109

[13] Saving J, Ponzer S, Enocson A, Mellstrand Navarro C. Distal radius fractures-regional variation in treatment regimens. PLoS One. 2018;13(11):e0207702. doi: 10.1371/journal.pone.0207702 30444926 PMC6239340

[14] Fanuele J, Koval KJ, Lurie J, Zhou W, Tosteson A, Ring D. Distal radial fracture treatment: what you get may depend on your age and address. J Bone Joint Surg Am. 2009;91(6):1313–9. doi: 10.2106/JBJS.H.00448 19487507 PMC2686132

[15] Walenkamp MMJ, Mulders MAM, Goslings JC, Westert GP, Schep NWL. Analysis of variation in the surgical treatment of patients with distal radial fractures in the Netherlands. J Hand Surg Eur Vol. 2017;42(1):39–44. doi: 10.1177/1753193416651577 27289051

[16] Benchimol EI, Smeeth L, Guttmann A, Harron K, Moher D, Petersen I, et al. The REporting of studies Conducted using Observational Routinely-collected health Data (RECORD) statement. PLoS Med. 2015;12(10):e1001885. doi: 10.1371/journal.pmed.1001885 26440803 PMC4595218

[17] Ludvigsson JF, Otterblad-Olausson P, Pettersson BU, Ekbom A. The Swedish personal identity number: possibilities and pitfalls in healthcare and medical research. Eur J Epidemiol. 2009;24(11):659–67. doi: 10.1007/s10654-009-9350-y 19504049 PMC2773709

[18] Statistics. Socialstyrelsen. 2023. Accessed June 8, 2023. https://www.socialstyrelsen.se/en/statistics-and-data/statistics/

[19] Steindel SJ. International classification of diseases, 10th edition, clinical modification and procedure coding system: descriptive overview of the next generation HIPAA code sets. J Am Med Inform Assoc. 2010;17(3):274–82. doi: 10.1136/jamia.2009.001230 20442144 PMC2995704

[20] Nordisk Medicinal-Statistisk Komit. NOMESCO classification of surgical procedures. Nordic Medico-Statistical Committee. 2010.

[21] Ludvigsson JF, Andersson E, Ekbom A, Feychting M, Kim J-L, Reuterwall C, et al. External review and validation of the Swedish national inpatient register. BMC Public Health. 2011;11:450. doi: 10.1186/1471-2458-11-450 21658213 PMC3142234

[22] Südow H, Sjödin L, Mellstrand Navarro C. Validity of distal radius fracture diagnoses in the Swedish National Patient Register. Eur J Med Res. 2023;28(1):335. doi: 10.1186/s40001-023-01314-0 37689700 PMC10492293

[23] Statistics Sweden. Statistikmyndigheten SCB. Accessed 2025 January 15. https://www.scb.se/en/

[24] Astrand J, Thorngren K-G, Tägil M. One fracture is enough! Experience with a prospective and consecutive osteoporosis screening program with 239 fracture patients. Acta Orthop. 2006;77(1):3–8. doi: 10.1080/17453670610045623 16534695

[25] Mellstrand Navarro C. Nationellt vårdprogram för behandling av distala radiusfrakturer. 2021. Accessed 2021 May 29. https://d2flujgsl7escs.cloudfront.net/external/Nationellt%20v%C3%A5rdprogram%20f%C3%B6r%20behandling%20av%20distala%20radiusfrakturer.pdf

[26] Schmidt V, Mellstrand Navarro C, Ottosson M, Tägil M, Christersson A, Engquist M, et al. Forecasting effects of “fast-tracks” for surgery in the Swedish national guidelines for distal radius fractures. PLoS One. 2022;17(2):e0260296. doi: 10.1371/journal.pone.0260296 35143508 PMC8830720

[27] Birkmeyer JD, Reames BN, McCulloch P, Carr AJ, Campbell WB, Wennberg JE. Understanding of regional variation in the use of surgery. Lancet. 2013;382(9898):1121–9. doi: 10.1016/S0140-6736(13)61215-5 24075052 PMC4211114

[28] Fanuele J, Koval KJ, Lurie J, Zhou W, Tosteson A, Ring D. Distal radial fracture treatment: what you get may depend on your age and address. J Bone Joint Surg Am. 2009;91(6):1313–9. doi: 10.2106/JBJS.H.00448 19487507 PMC2686132

