# Supplementary material for: Fracture fixation in the hand and wrist: A 16-year population-based study of 56 163 patients from the Swedish National Patient Register
Source: PLoS One. 2025 Sep 3;20(9):e0330116. doi: 10.1371/journal.pone.0330116 (PMC12407425; doi:10.1371/journal.pone.0330116)
Supplement: S1 Table — (DOCX) [file pone.0330116.s001.docx]

**S1 Table:** Adjusted R2 values for tested regression models.

| **Model** | **Adjusted R2** | **Best fit?** |
| --- | --- | --- |
| Linear | 0.41 | No |
| Exponential | 0.37 | No |
| Logarithmic | 0.41 | No |
| Polynomial (2nd order) | 0.73 | Yes |
| Power | 0.37 | No |
