# Supplementary material for: Fracture fixation in the hand and wrist: A 16-year population-based study of 56 163 patients from the Swedish National Patient Register
Source: PLoS One. 2025 Sep 3;20(9):e0330116. doi: 10.1371/journal.pone.0330116 (PMC12407425; doi:10.1371/journal.pone.0330116)
Supplement: Attachment — Submitted filename: Point-by-point response to reviewers.docx [file pone.0330116.s002.docx]

**Manuscript**

**Response to Reviewers**

Dear Editor,

The authors thank you for considering the present manuscript. We appreciate the effort that the reviewers dedicated to improving the manuscript and are grateful for the insightful comments on and valuable improvements to the paper.
We have addressed the reviewers’ remarks below and the corresponding changes have been made in the revised manuscript.

The authors hope that you will find the manuscript suitable for future publication in PLOS ONE.

Best regards,

Viktor Schmidt
Corresponding author

**Reviewer #1:**
Thank you for your review and good input.

*There is a minor typo on Page 10, Line 243. The word "to" should come before "enable".*

**Author response:** Thank you, we have edited accordingly.

*I have just one other suggestion for the authors to consider that was prompted by their comments in the Limitations section of the paper, noting that bone quality may influence treatment decisions and outcomes. I have read that the Swedish National Patient Register (NPR) does not include data on prescription medications. This information is held on the Swedish Prescribed Drug Register (SPDR) as the authors will know. A brief literature search identified several linkage studies combining data from both registries such as a 2020 study on surgical site infections after distal radius fracture surgery (see https://bmcmusculoskeletdisord.biomedcentral.com/articles/10.1186/s12891-020-03822-0) and a 2021 study that explored associations between hormonal contraception and antidepressant use (see* [*https://bmjopen.bmj.com/content/11/10/e049553*](https://bmjopen.bmj.com/content/11/10/e049553)*).*

*It would be interesting to see an NPR-SPDR linkage study to evaluate trends in prescription of osteoporosis specific medications among patients undergoing fracture fixation of the wrist, or indeed other types of fragility fractures occurring among people aged 50 years and older.*

*In the event that the authors have any intentions in the future to undertake linkage studies with data from the NPR and SPDR, readers of their publication may be interested to know of this future direction.*

**Author response:** Thank you, this would be an interesting study to conduct. We do not have any such plans, but it might be something for the future!

**Reviewer #2:**
Thank you for your thorough review and comments. *This is a valuable, well-structured, and timely manuscript that analyzes long-term national data on hand and wrist fracture fixation procedures in Sweden. The use of a large, population-based dataset over a 16-year span provides robust epidemiological insights, particularly regarding temporal trends, regional variation, and demographic characteristics. The topic is highly relevant for both clinical orthopaedics and healthcare planning, especially in light of aging populations and increasing surgical intervention rates.
The manuscript is generally well-written, the data are clearly presented, and the conclusions are supported by the results. However, several areas require further clarification, elaboration, and refinement. Below I outline specific strengths and areas for improvement.*

*Major Strengths
Comprehensive National Dataset: The use of the Swedish National Patient Register (NPR) ensures a high degree of population representativeness and statistical power. The inclusion of 56,163 patients provides a strong foundation for trend analysis.
Public Health Relevance: The findings have clear implications for health resource allocation, surgical training, and guideline implementation. The observed increase in fixation procedures among elderly women and regional disparities are important from a health equity perspective.
Use of Predictive Modelling: The attempt to forecast future surgical trends through regression modelling adds value, particularly for anticipating future healthcare demands.*

**Author response:** Thank you.

*Methodological Transparency of Predictive Modelling
While multiple regression models (linear, exponential, etc.) were tested, the manuscript does not provide sufficient detail on model selection criteria. The reporting of R² values is not enough—please include a table comparing AIC/BIC or adjusted R² values for each model type to support your choice of best-fitting models. Additionally, assumptions behind the regression analysis (e.g., linearity, independence, stationarity) should be briefly discussed, and model validation (if any) should be mentioned.*

**Author response:** We have now added a table with adjusted R2 values for each model type. We have also added assumptions behind the regression modelling.

*Data Limitations and Potential Biases
The study relies on procedural coding (NDJ69), but it is unclear whether this code uniformly represents only open reduction and internal fixation with plates and screws. Could this include other modalities or anatomical sites beyond the wrist/hand? Please clarify the specificity of this code. The manuscript mentions possible regional differences in clinical practices. However, another likely contributor is variation in data reporting or coding fidelity across regions. Consider discussing this potential source of bias and its impact on results.*

**Author response:** Thank you. We have edited the discussion.

**L255 edited to:** The accuracy of the NPR depends on consistent and precise reporting from healthcare providers. Variations in coding practices, potential miscoding, transcription errors, underreporting, and missing data may influence data quality, as is typical in register-based studies. These variations could differ between regions. The specific procedural code NDJ69 represents open reduction and internal fixation of a fracture in the wrist or hand with plates and screws. It does not include other modalities or anatomical sites.

*Interpretation of Regional Disparities
While geographic variations are noted, explanations remain somewhat speculative. A deeper discussion of possible factors—such as socioeconomic differences, distribution of orthopedic specialists, hospital capacity, or urban–rural demographics—would significantly enhance the interpretive strength. Consider supplementing Figure 3 with quantitative data (e.g., in a supplementary table) for transparency and replicability.*

**Author response:** Thank you, we have edited accordingly.

*Impact of National Guidelines (2021)
The paper discusses the introduction of Swedish national guidelines in 2021, but does not quantitatively analyze their impact. Given the timeline (2008–2023), this represents an opportunity for an interrupted time series (ITS) analysis or stratification of data before/after 2021 to demonstrate policy impact more convincingly.*

**Author response:** Thank you, this would have been an interesting analysis to do if we had more granular data, unfortunately this paper analyses hand and wrist fractures (NDJ69) meanwhile the national guidelines only pertain to distal radius fractures. Thus, the guidelines could have affected the study but performing an ITS on this cohort would not be a fair analysis potentially leading to unjustified conclusions.

*Language and Style
Overall, the manuscript is well-written, though some sections would benefit from stylistic tightening.Ensure consistent use of English spelling conventions (e.g., “favourable” vs. “favorable”).The phrase “elderly women” appears frequently—consider using more neutral phrasing such as “women aged 65 years and older” in some contexts.*

**Author response:** Thank you, we have replaced the phrase “elderly women” according to your suggestions. However, we found that we have used “favourable” throughout and will keep it as we have written the rest of the manuscript in British English.

*Figures
Figure 1 caption should clearly define “All fractures” and clarify if it includes non-surgical management.Color schemes in Figure 3 could be made more colorblind-friendly and readable.
Please indicate the statistical significance of trends (p-values or confidence intervals) directly in the figures where appropriate.*

**Author response:** Thank you, we have edited accordingly. Figure 1 caption reads: Trends in hand and wrist fracture fixation with plates and screws, 2008–2023. “All fractures” represent those treated with all modalities (non-operative included). The other three trend lines (Men, Women and All) represent only fixation using plates and screws.

Figure 3 has been made colorblind-friendly.

We have added p-values to Figure 2.

*Ethical Considerations
Although the study uses publicly available data and is exempt from ethical approval, a brief statement on data anonymization and privacy safeguards would be helpful.*

**Author response:** Thank you, we have edited accordingly.

**L151 changed to:** This study relied exclusively on open-access data (which is anonymized and aggregated to avoid small-cell risks) and was thus exempt from ethical review as Swedish law does not require ethical permit for the study of group level open access data.

*EDITORIAL COMMENTS:

1. Please ensure that your manuscript meets PLOS ONE's style requirements, including those for file naming. The PLOS ONE style templates can be found at*

*https://journals.plos.org/plosone/s/file?id=wjVg/PLOSOne_formatting_sample_main_body.pdf and*

[*https://journals.plos.org/plosone/s/file?id=ba62/PLOSOne_formatting_sample_title_authors_affiliations.pdf*](https://journals.plos.org/plosone/s/file?id=ba62/PLOSOne_formatting_sample_title_authors_affiliations.pdf)

**Author response:** Thank you, we have gone through the templates.

*2. Thank you for uploading your study's underlying data set. Unfortunately, the repository you have noted in your Data Availability statement does not qualify as an acceptable data repository according to PLOS's standards. At this time, please upload the minimal data set necessary to replicate your study's findings to a stable, public repository (such as figshare or Dryad) and provide us with the relevant URLs, DOIs, or accession numbers that may be used to access these data. For a list of recommended repositories and additional information on PLOS standards for data deposition, please see* [*https://journals.plos.org/plosone/s/recommended-repositories*](https://journals.plos.org/plosone/s/recommended-repositories)*.*

**Author response:** We have uploaded the data set. We have uploaded the data to: https://doi.org/10.6084/m9.figshare.29389934.v1

DOI: 10.6084/m9.figshare.29389934

*3. We note that Figure 3 n your submission contain [map/satellite] images which may be copyrighted. All PLOS content is published under the Creative Commons Attribution License (CC BY 4.0), which means that the manuscript, images, and Supporting Information files will be freely available online, and any third party is permitted to access, download, copy, distribute, and use these materials in any way, even commercially, with proper attribution. For these reasons, we cannot publish previously copyrighted maps or satellite images created using proprietary data, such as Google software (Google Maps, Street View, and Earth). For more information, see our copyright guidelines: http://journals.plos.org/plosone/s/licenses-and-copyright.*

*We require you to either (1) present written permission from the copyright holder to publish these figures specifically under the CC BY 4.0 license, or (2) remove the figures from your submission:*

*1. You may seek permission from the original copyright holder of Figure 3 to publish the content specifically under the CC BY 4.0 license.*

*We recommend that you contact the original copyright holder with the Content Permission Form (http://journals.plos.org/plosone/s/file?id=7c09/content-permission-form.pdf) and the following text:*

*“I request permission for the open-access journal PLOS ONE to publish XXX under the Creative Commons Attribution License (CCAL) CC BY 4.0 (http://creativecommons.org/licenses/by/4.0/). Please be aware that this license allows unrestricted use and distribution, even commercially, by third parties. Please reply and provide explicit written permission to publish XXX under a CC BY license and complete the attached form.”*

*Please upload the completed Content Permission Form or other proof of granted permissions as an ""Other"" file with your submission.*

*In the figure caption of the copyrighted figure, please include the following text: “Reprinted from [ref] under a CC BY license, with permission from [name of publisher], original copyright [original copyright year].”*

*2. If you are unable to obtain permission from the original copyright holder to publish these figures under the CC BY 4.0 license or if the copyright holder’s requirements are incompatible with the CC BY 4.0 license, please either i) remove the figure or ii) supply a replacement figure that complies with the CC BY 4.0 license. Please check copyright information on all replacement figures and update the figure caption with source information. If applicable, please specify in the figure caption text when a figure is similar but not identical to the original image and is therefore for illustrative purposes only.*

*The following resources for replacing copyrighted map figures may be helpful:*

*USGS National Map Viewer (public domain): http://viewer.nationalmap.gov/viewer/*

*The Gateway to Astronaut Photography of Earth (public domain): http://eol.jsc.nasa.gov/sseop/clickmap/*

*Maps at the CIA (public domain): https://www.cia.gov/library/publications/the-world-factbook/index.html and https://www.cia.gov/library/publications/cia-maps-publications/index.html*

*NASA Earth Observatory (public domain): http://earthobservatory.nasa.gov/*

*Landsat: http://landsat.visibleearth.nasa.gov/*

*USGS EROS (Earth Resources Observatory and Science (EROS) Center) (public domain): http://eros.usgs.gov/#*

*Natural Earth (public domain):* [*http://www.naturalearthdata.com/*](http://www.naturalearthdata.com/)

**Author response:** We have Illustrator 2025 (29.5.1) to draw our own maps and colour them.

*4. Please review your reference list to ensure that it is complete and correct. If you have cited papers that have been retracted, please include the rationale for doing so in the manuscript text, or remove these references and replace them with relevant current references. Any changes to the reference list should be mentioned in the rebuttal letter that accompanies your revised manuscript. If you need to cite a retracted article, indicate the article’s retracted status in the References list and also include a citation and full reference for the retraction notice.*

**Author response:** We have reviewed the reference list.
